# To assure aviation safety: the pilot fatigue detection based on short-term multimodal physiological signals

**DOI:** 10.3389/fnhum.2026.1743936

**Published:** 2026-02-03

**Authors:** Kai Chen, Jiming Liu, Jiamei Zhu, Yan Xu, Lin Zhang, Zhenxing Gao

**Affiliations:** 1 East China Personnel Medical Appraisal Center, Civil Aviation Shanghai Hospital, Shanghai, China; 2 College of Civil Aviation, Nanjing University of Aeronautics and Astronautics, Nanjing, China; 3 College of General Aviation and Flight, Nanjing University of Aeronautics and Astronautics, Nanjing, China

**Keywords:** electrocardiogram, electroencephalogram, fatigue detection, feature fusion, feature selection, flight simulator training

## Abstract

Pilot fatigue detection based on physiological signals is practical for aviation safety. Current methods face challenges in balancing the high computational cost of deep learning models with robust accuracy, especially when integrating short-term multimodal physiological signals. To address these challenges, this paper proposes a framework for fast, accurate, and robust pilot fatigue detection by fusing features from electroencephalogram (EEG) and electrocardiogram (ECG) signals. The primary novelty of this work lies in a streamlined selection and classification strategy that overcomes the intrinsic limitations of Heart Rate Variability (HRV) analysis in short (2-s) segments while maintaining competitive accuracy at a drastically lower training cost. Specifically, by utilizing statistical ECG features, which are then integrated with EEG markers through a two-stage ANOVA-SVM feature selection process. The optimized, low-dimensional feature set is then classified using an XGBoost model. Evaluated on data from 32 pilots, the framework demonstrated robust generalization with an accuracy of 88.42% in rigorous cross-subject cross-validation, significantly outperforming our previous EEG-only ASFT-Transformer. While standard cross-clip validation yielded a higher accuracy of 98.36%, the cross-subject metric highlights the model's potential utility for unseen individuals. Crucially, the framework achieves this performance with an average training time of only 39.3 s, a drastic reduction compared to mainstream deep learning models. By striking a balance between accuracy, generalization, and efficiency, this study presents a promising and feasible approach for objective pilot fatigue management.

## 1 Introduction

A persistent challenge in aviation safety is pilot fatigue, which directly impairs an operator's reaction speed, information integration, and decision-making capabilities (Hamann and Carstengerdes, 2023). Although extensive research has identified the roots of fatigue in sleep deprivation, high operational workload, and circadian misalignment (Mannawaduge et al., 2024; Berberich and Leitner, 2017; Marcus and Rosekind, 2017; Wingelaar-Jagt et al., 2021), a fundamental problem remains: human physiology dictates that optimal functioning relies on adequate nightly sleep, meaning fatigue cannot be completely eradicated (International Civil Aviation Organization, 2020). In this context, establishing a scientific and effective pilot fatigue management system is of paramount importance for ensuring aviation safety.

The International Civil Aviation Organization (ICAO) has published the latest version of Doc 9966, offering a guiding framework for pilot fatigue management (International Civil Aviation Organization, 2020). This document's principles have been adopted by major aviation authorities (Federal Aviation Administration, 2013; European Union Aviation Safety Agency, 2012; Civil Aviation Administration of China, 2021). Specifically, operators are strongly advised to evaluate pilot states based on continuous monitoring and data analysis. For data collection, although self-reporting scales are often proposed (Maclean et al., 1992; Kaida et al., 2006), fatigue indicators derived from this self-reporting process are largely unreliable in practice. Pilots may struggle to accurately evaluate their own condition against multiple fatigue levels (Guo et al., 2025; Gläsener et al., 2023), and more seriously, some may conceal their fatigue for certain reasons (such as failing to meet the flight duration requirements), thereby endangering aviation safety (Jun-Ya and Rui-Shan, 2023; Kioulepoglou et al., 2024; Brezonakova, 2023). Consequently, Doc 9966 identifies the collection and analysis of physiological data as a superior approach for fatigue management.

Unlike subjective self-reports, physiological data, including Electroencephalogram (EEG) (van Weelden et al., 2022), Electrocardiogram (ECG) (Guo et al., 2025), Electromyogram (EMG) (Naim et al., 2022), and Electrooculogram (EOG) (Faust et al., 2018), provides objective metrics for assessing the functional state of the human body. Utilizing these modalities for fatigue detection in pilots and drivers is a well-established methodology. Research has demonstrated that EEG frequency bands (specifically δ, θ, α, and β) are strongly correlated with workload, fatigue levels, and other functional states (Eoh et al., 2005). Regarding cognitive load, increased task difficulty typically results in elevated θ power (especially in frontal regions) and suppressed α power across frontal, central, and parietal areas, whereas β power exhibits mixed responses (decreasing in parietal regions or generally increasing) (Chikhi et al., 2022). Conversely, the onset of mental fatigue triggers distinct spectral shifts: δ notably increases; θ and α power both rise in frontal and parietal regions (with α also increasing in occipital areas) (Borghini et al., 2014). Meanwhile, β power responses remain variable under fatigue, with studies reporting either overall power decreases or parietal increases (Hamann and Carstengerdes, 2022). For ECG, the Heart Rate Variability (HRV) component extracted from ECG is particularly notable, as it reflects autonomic nervous system activity, is highly correlated with fatigue (Wang et al., 2018; Chen et al., 2024; Wang F. et al., 2024; Gao et al., 2025) and has proven effective in tasks like detecting sleep states (Piotrowski and Szypulska, 2017) and classifying physical fatigue (Ni et al., 2022). Regarding EMG, muscle activity typically exhibits a decrease in signal amplitude and a spectral shift toward lower mean power frequencies (muscle fatigue) in postural muscles, reflecting the reduced muscle tone and motor coordination associated with drowsiness (Naim et al., 2022). Additionally, EOG indicators serve as reliable detectors of visual vigilance, where the onset of fatigue is typically marked by significantly increased blink duration and frequency, alongside slower saccadic movements due to reduced oculomotor control (Faust et al., 2018).

Notably, EEG is often favored for workload and fatigue evaluation (Blanco et al., 2018; Luo et al., 2019; Dehais et al., 2019; Wang Y. et al., 2024). Its prominence stems from its ability to directly reflect brain activity (Zhang et al., 2023), making it highly suitable for end-to-end detection of human functional states via artificial neural networks, which bypasses the need for prior knowledge (Huang et al., 2022). Consequently, several deep learning architectures are widely applied to EEG data. Convolutional Neural Networks (CNNs), for instance, are commonly used for cognitive workload assessment (Jiao et al., 2018), emotion recognition (Shen et al., 2023), and fatigue detection (Gao et al., 2019). In parallel, Recurrent Neural Networks (RNNs), notably the Long Short-Term Memory (LSTM) (Tang et al., 2021; Cao et al., 2024) and Bi-LSTM (Mehmood et al., 2023), are increasingly adopted for processing its temporal sequences. Furthermore, research aiming to enhance spatio-temporal feature extraction has yielded favorable results from hybrid structures, including the CNN-LSTM (Pan et al., 2023), LGNet (Wang Y. et al., 2024), and MATCNT (Jia et al., 2023).

Despite its promise, end-to-end deep learning for fatigue detection is computationally intensive due to the massive volume of raw EEG data (Zheng and Lu, 2017), carrying risks of prolonged training times and technical challenges like gradient instability and overfitting. To mitigate these issues, researchers often employ feature extraction to lessen data complexity and boost efficiency (Zhang et al., 2023). This has led to successful models using entropy features (Luo et al., 2019; Gao et al., 2024a,b) as well as various time-domain, frequency-domain, and brain-network features (Dehais et al., 2019; Zhang et al., 2023; Liu et al., 2021; Wang et al., 2025; Jebelli et al., 2018; Zhou et al., 2023; Wang et al., 2021; Fu et al., 2020). However, this introduces the curse of dimensionality. While feature selection is the standard solution, existing strategies often fail to strike a balance between computational speed and physiological validity. Wrapper methods, such as those based on recursive feature elimination or genetic algorithms, incur prohibitive computational costs due to iterative model retraining, making them unsuitable for rapid, deployment-ready screening (Wang et al., 2021). Conversely, simple filter methods are computationally efficient but often overlook complex dependencies between multimodal features, potentially discarding variables that are weak individually but predictive in combination (Liu et al., 2021). Furthermore, while emerging deep learning approaches perform implicit feature extraction, they typically operate as “black boxes” (Faust et al., 2018). They lack the physiological interpretability required to explicitly identify which specific channels and features drive the fatigue classification (Zhang et al., 2023). Consequently, a critical gap remains: how to efficiently identify a feature subset that is not only statistically discriminative but also biologically meaningful.

To bridge this gap, feature selection must be grounded in neuroergonomic principles rather than relying solely on data-driven metrics. Physiologically, pilot fatigue is characterized by distinct spectral and spatial alterations, specifically a global increase in slow-wave activity (δ and θ bands) and elevated Alpha power in occipital and parietal regions, reflecting diminished visual attention (Jing et al., 2020; Borghini et al., 2014). Unlike prior approaches that either obscure these mechanisms within opaque deep neural networks (Gao et al., 2019) or rely on rigid, pre-defined manual selection, our framework employs a transparent, data-driven strategy. By systematically screening features through the ANOVA-SVM pipeline, we objectively isolate the most fatigue-sensitive markers, specifically confirming the predominance of occipital channels without prior assumptions (as detailed in Section 3.2). This approach not only aligns engineering design decisions with established neurophysiological mechanisms but also ensures the model's robustness and generalization across different subjects.

Concurrently, a growing trend involves constructing multimodal features by integrating ECG with EEG (Mu et al., 2024; Gao et al., 2025), leveraging the ECG's own utility as a viable signal for assessing mental and physical states (Guo et al., 2025; Chen et al., 2024; Wang F. et al., 2024). While both modalities are informative individually, the most significant advantages emerge from their fusion. Research has consistently demonstrated that combining EEG and ECG signals enhances overall detection accuracy and robustness. For instance, (Awais et al. 2017) demonstrated improved accuracy using an SVM-based fusion of the two signals. Similarly, (Mu et al. 2024) achieved 94% accuracy by designing a novel gating method to adaptively fuse ECG and HRV features. (Du et al. 2023) further confirmed this, using a Product Fuzzy Convolutional Network (PFCN) with a dedicated subnetwork for fusion, which resulted in superior robustness and accuracy. These findings collectively validate that a multimodal EEG-ECG approach can overcome the limitations of single-signal analysis.

Although significant progress has been made, several key challenges persist for practical application in pilot fatigue management:

(1) Real-time fatigue monitoring of pilots based on physiological signals is difficult. Extended wear of standard EEG and ECG sensors may introduce physical discomfort or cognitive interference, potentially distracting pilots from critical flight tasks. Furthermore, the logistical complexity of equipping cockpits with real-time signal processing and transmission hardware presents a substantial barrier to scalable, daily deployment, effectively limiting the practical utility of such real-time systems.

(2) Short-term ECG signals have limitations in fatigue detection. In the context of (1), a practical and objective pilot fatigue management solution is to use physiological signals to determine whether pilots are already fatigued before flight, which means that the acquisition time of physiological signal data for each pilot will be short. However, a too short signal may not accurately capture complete physiological information, thus affecting the reliability of fatigue assessment. Crucially, traditional frequency-domain HRV analysis, which requires at least 5 min of data to reliably reflect autonomic nervous system (ANS) regulation, is inapplicable to short segments (Chen et al., 2024; Sun et al., 2024).

(3) It is challenging for fatigue detection based on bimodal EEG-ECG to satisfy the requirements of small input samples, fast model training and high recognition accuracy. Although many studies have been able to achieve more accurate fatigue detection based on EEG signals of a few seconds (Zheng and Lu, 2017; Gao et al., 2024a,b; Wang et al., 2025; Li et al., 2024), effectively fusing these with short-term ECG signals without inflating computational cost or overfitting remains difficult. Furthermore, the key to achieve fatigue detection with short model training time and high recognition accuracy lies in the co-optimization of both the feature selection and the classification model itself.

To avoid the practical limitations of in-flight monitoring, this study proposes a strategic framework tailored for pre-flight fatigue detection, offering a feasible alternative for daily safety management. Building upon our previous exploration of EEG-based pre-flight fatigue detection (Liu et al., 2025), this study optimizes the framework by incorporating short-term ECG features. Although the standard time period for HRV analysis is usually 5 min, research indicates that fatigue-induced shifts in sympathetic and parasympathetic balance manifest not only in heart rate intervals but also in the morphological complexity and statistical distribution of the raw ECG signal (Butkevičiūtė et al., 2022; Mu et al., 2024). Statistical features, such as variance, skewness, and kurtosis, quantify the signal's deviation from a normal distribution and its transient instability. These statistics serve as robust physiological proxies for the immediate autonomic perturbations and non-linear complexity changes associated with mental fatigue, even within restricted time windows (Guo et al., 2025). Therefore, this study integrates ECG statistical features with EEG data to train a computationally efficient XGBoost classifier. This approach offers a dual advantage: it enhances the accuracy and robustness of fatigue detection while significantly reducing model training time.

## 2 Methodology

### 2.1 Analytical framework overview

**Figure 1:**
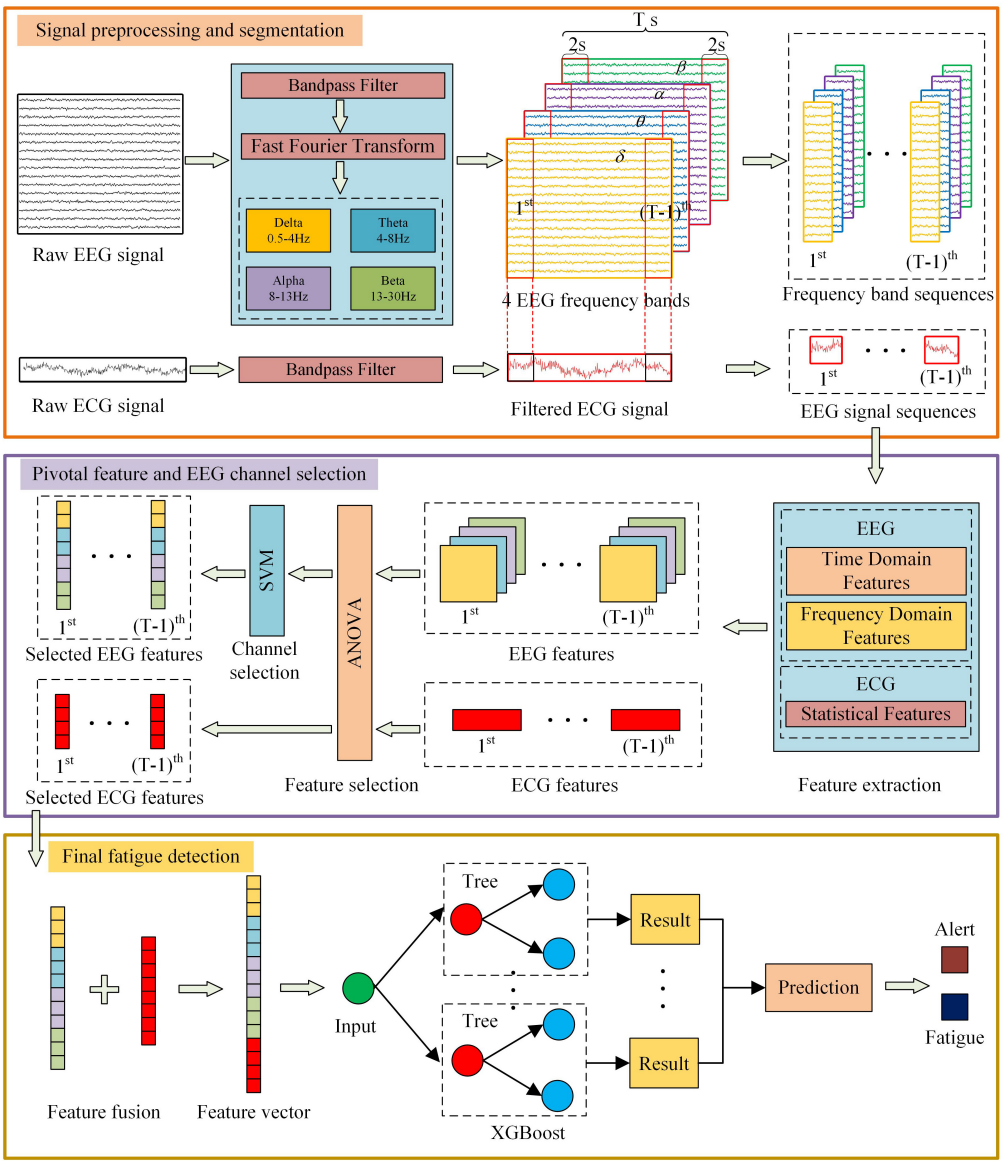
Schematic overview of the proposed pilot fatigue detection framework. The pipeline consists of three sequential stages: (1) Signal preprocessing and segmentation of EEG/ECG data, (2) Pivotal feature selection using ANOVA and EEG channel selection using SVM-based AUC ranking, and (3) Final fatigue classification using the XGBoost model. With permission from Liu et al. (2025).

The analytical framework for pilot fatigue detection is depicted in Figure 1. This process is structured into three sequential stages: (1) signal preprocessing and segmentation, (2) pivotal feature and EEG channel selection, and (3) final fatigue classification. Each component is detailed in the subsequent sections.

### 2.2 Experimental dataset and procedure

#### 2.2.1 Participant characteristics

To rigorously mitigate potential confounds and ensure that the detected physiological shifts were primarily driven by task-induced fatigue rather than sleep deprivation or circadian misalignment, strict inclusion criteria were enforced. Specifically, all participants were required to: (1) maintain at least 8 h of nightly sleep for the two days preceding the experiment; (2) abstain from alcohol, caffeine, and any drowsiness-inducing medications for 48 h prior to the study; and (3) verify the absence of any neurological or psychiatric history. Regarding the timing of the experiment, simulation sessions were scheduled between 06:00 and 08:00 (local time) contingent on the airline's operational roster. While precise start times varied slightly due to scheduling logistics, strictly maintaining this consistent morning window minimized the impact of circadian phase differences across subjects. Following a complete briefing of the experimental procedure, written informed consent was obtained from each pilot. The study protocol adhered to the principles of the Declaration of Helsinki and received ethical approval from the Civil Aviation Shanghai Hospital, China.

#### 2.2.2 Fatigue induction procedure

**Figure 2:**
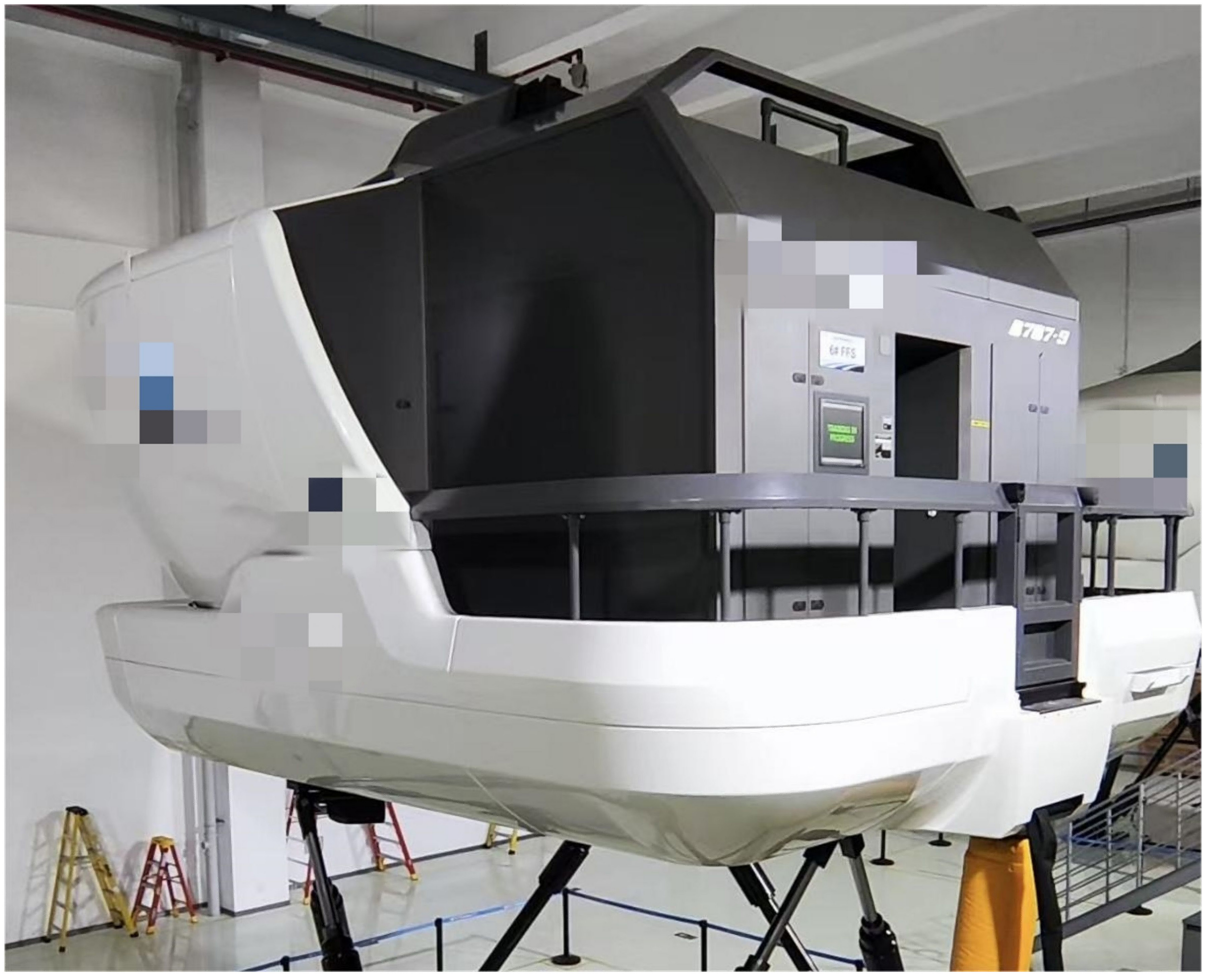
Illustration of the Boeing 787 simulator. This high-fidelity simulator was used to conduct the 6-h flight training mission. The realistic operational environment ensures that the induced fatigue reflects actual flight conditions rather than simple laboratory drowsiness.

To procure physiological data representing alert and fatigue states, we collected EEG and ECG recordings from each pilot immediately before (pre-training) and after (post-training) their standard regular simulator training. This training, which lasted approximately 6 h, was executed in a Boeing 787 Level D full-flight simulator at the airline's Flight Training Center (Figure 2). The training procedure, grounded in Evidence-Based Training (EBT) principles (International Civil Aviation Organization, 2013), lasted a total of about 6 h. Each training session paired one captain with one first officer under the supervision of a flight instructor. The procedure was divided into four main components:

(1) Pre-flight Briefing (1 h): The instructor outlined the training scenarios, maneuver checks, and evaluation criteria.

(2) Scenario-Based Training (SBT) (2 h): The crew managed 1–2 complete flights involving various anticipated and unanticipated challenges, such as engine failures or adverse weather conditions.

(3) Maneuver-Based Checks (MBC) (2 h): The crew performed specific maneuvers, including various subjects, such as take-off with crosswind, non-precision approach and landing with one engine inoperative.

(4) Post-flight Debriefing (1 h): The instructor provided objective feedback on crew performance to identify areas for improvement.

**Figure 3:**
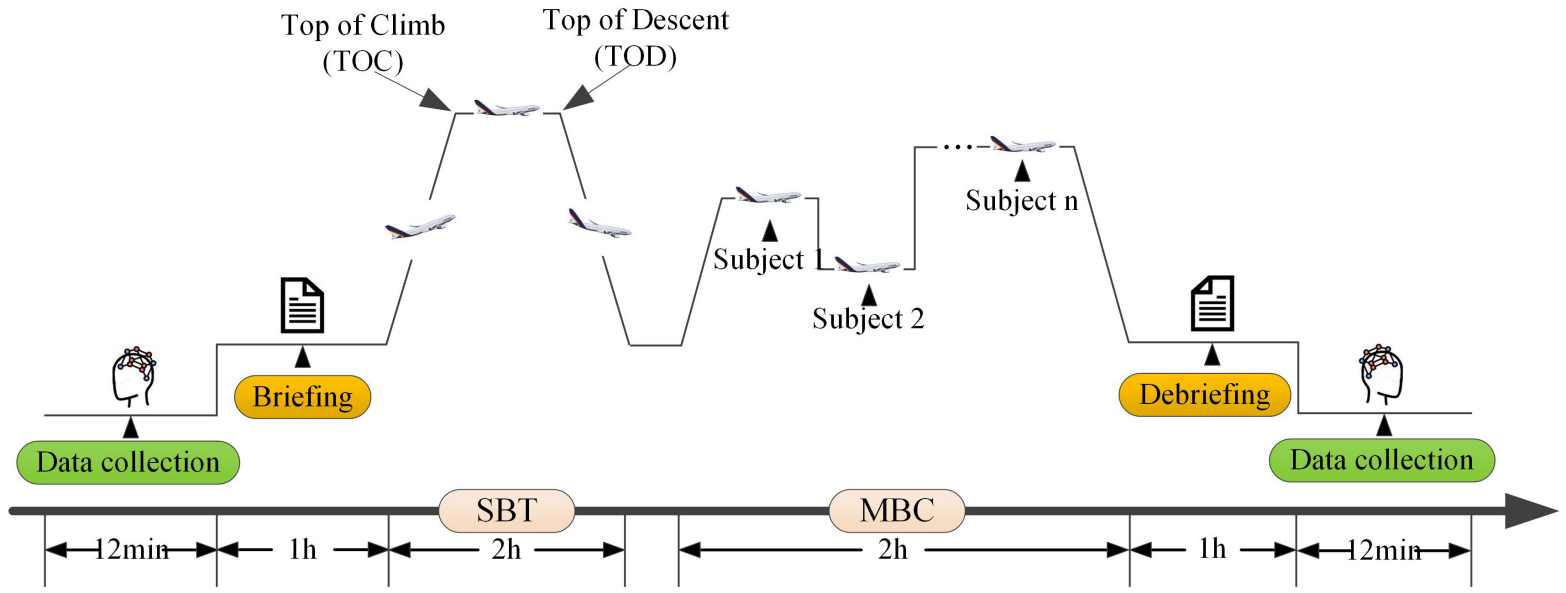
Schematic of the fatigue induction procedure. The 6-h procedure follows EBT, principles, including Pre-flight Briefing, SBT, MBC and Post-flight Debriefing. Physiological data are collected before (pre-training) and after (Post-training) the procedure to capture alert and fatigue states, respectively.

Figure 3 illustrates this entire training procedure. Physiological data were recorded continuously, and the labeling strategy was defined as follows: the data collected during the first 10 min of the session was labeled as the Alert State, while the data from the last 10 min was labeled as the Fatigue State. This labeling strategy was grounded in the physiological effects of prolonged cognitive exertion to avoid the potential variability and subjectivity inherent in self-reported ratings. Previous aviation physiology research suggests that continuous performance of demanding simulated flight tasks typically induces significant mental fatigue and EEG slow-wave activity after 2–4 h (Guo et al., 2025; Wang et al., 2025; Borghini et al., 2014; Caldwell et al., 2009; Mangin et al., 2022). In this study, the 6-h duration provided a substantial operational window beyond these reported thresholds. Consequently, we adopted the assumption that participants had transitioned into a fatigued state by the end of the session.

#### 2.2.3 Physiological signal acquisition

**Figure 4:**
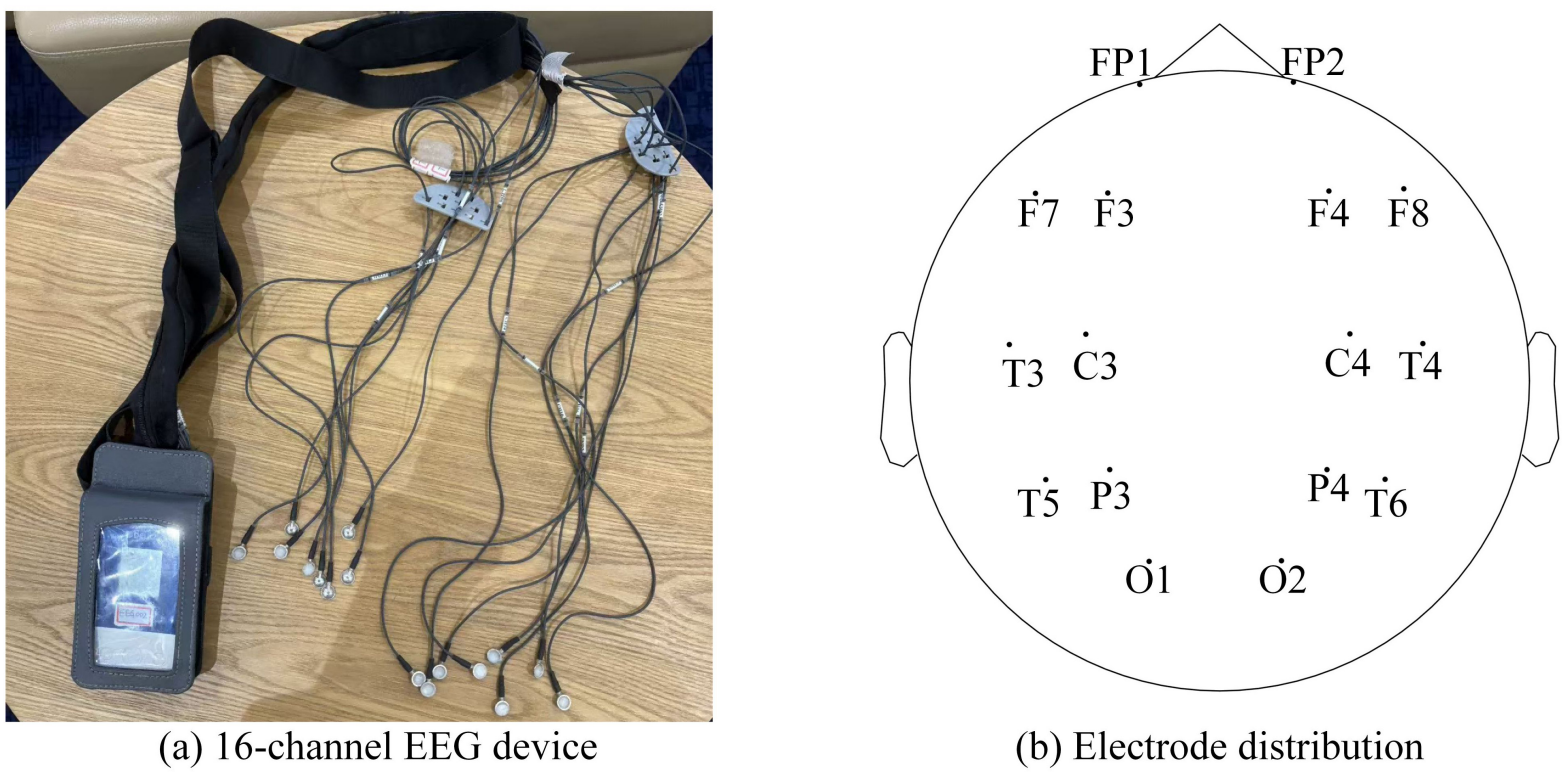
Illustration of the EEG device. **(a)** The 16-channel Delica AEEG-3202 device with standard Ag/AgCl wet electrodes. **(b)** The electrode distribution on the scalp follows the international 10–20 system, covering Frontal (F), Central (C), Parietal (P), Temporal (T), and Occipital (O) regions. Image adapted with permission from Liu et al. (2025).

Pilot EEG signals were collected using a 16-channel Delica AEEG-3202 electroencephalograph (Shenzhen Delica Medical Equipment Co., Ltd., China). This system, comprising a compact control box and 16 standard Ag/AgCl wet disc electrodes (Figure 4a), provided high-quality data at a 500 Hz sampling rate. The 16 electrodes were positioned on the scalp according to the standard international convention, as shown in Figure 4b (FP: Frontopolar, F: Frontal, C: Central, P: Parietal, T: Temporal, O: Occipital; odd/even numbers denote left/right hemispheres).

**Figure 5:**
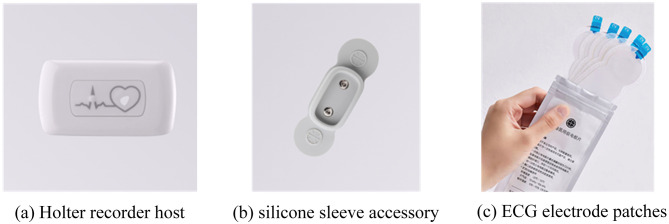
Illustration of the ECG device. The single-channel Mi-Rhythm Holter recorder includes **(a)** the host unit, **(b)** a protective silicone sleeve, and **(c)** disposable electrode patches. This compact design allows for unobtrusive recording of cardiac activity.

Pilot ECG signals were collected using a single-channel Mi-Rhythm Holter ECG recorder (Shenzhen Ruikang Hongye Technology Development Co., Ltd., China) at a sampling rate of 250 Hz. The ECG device consists of a Holter recorder, silicone sleeve, and disposable electrode patches, as shown in Figure 5.

**Figure 6:**
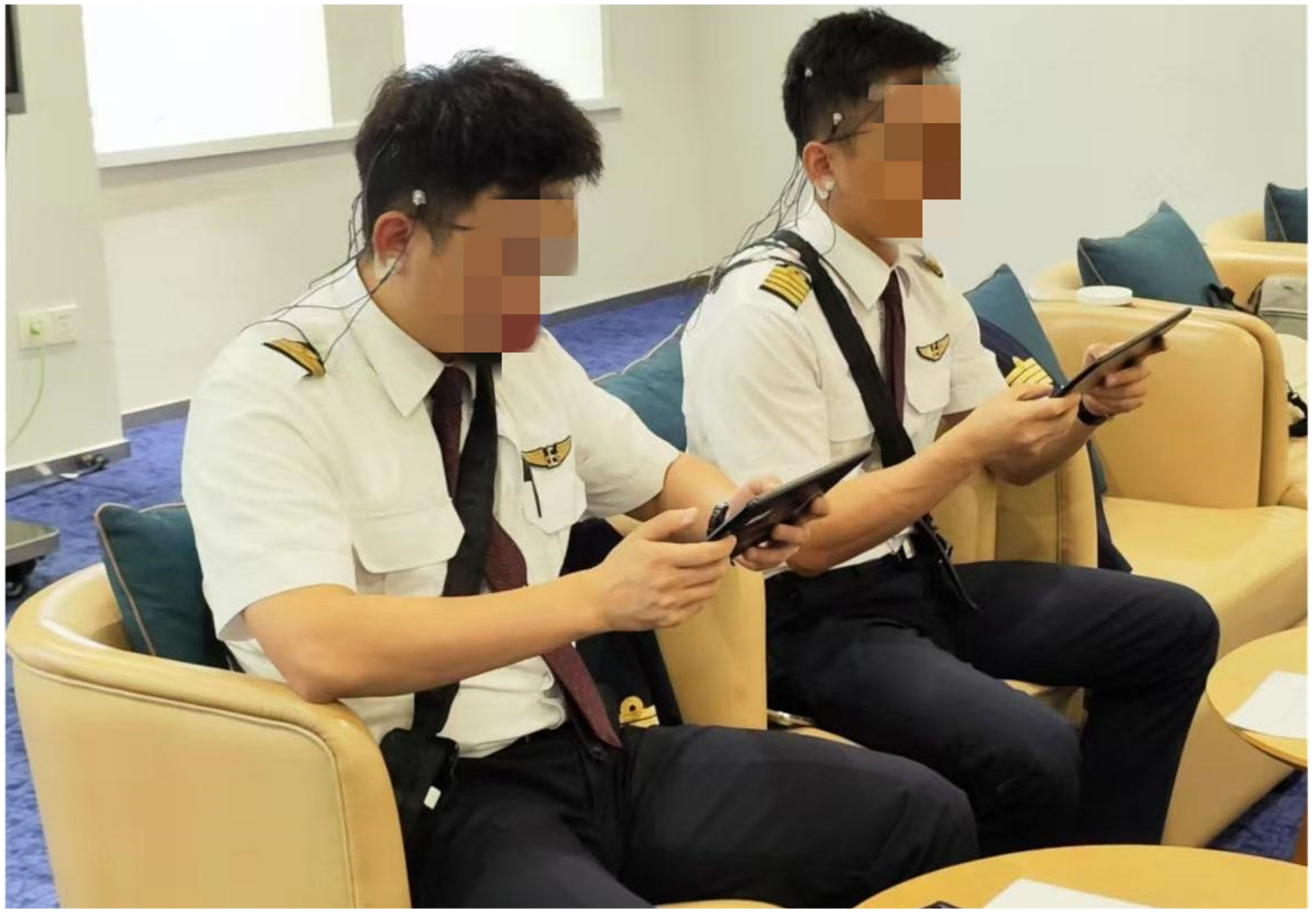
Schematic diagram of collecting physiological signals for pilots. Data collection was conducted in a quiet, controlled room while pilots remained in a seated, eyes-open resting state. This setup minimizes movement artifacts and ensures the signals reflect mental fatigue rather than sleep onset. Image adapted from Figure 4, Liu et al. (2025).

To minimize participant discomfort and facilitate rapid detection, synchronous EEG and ECG data were recorded for approximately 12 min during both the pre-training and post-training sessions (Figure 6). The data collection was conducted in a quiet, controlled environment where participants were seated comfortably. Crucially, to mitigate behavioral confounds and simulate the visual vigilance required in operational settings, all pilots were instructed to maintain an eyes-open resting state to ensure they remained awake and strictly adhered to the protocol, thereby ensuring the signals reflected mental fatigue rather than drowsiness-induced sleep onset.

For a balanced and standardized analysis, only the initial 10 min of each recording were retained for processing. Given that the EEG and ECG signals were acquired via independent systems, hardware-based synchronization was not employed. Instead, temporal alignment was strictly performed post-acquisition. Specifically, the data streams from both modalities were synchronized by manually aligning their absolute recording timestamps. This procedure ensured that the retained 10-min segments for both EEG and ECG corresponded to the exact same temporal window for each subject.

### 2.3 Signal preprocessing and segmentation

To evaluate the robustness of the proposed framework in realistic, resource-constrained aviation environments, the raw EEG and ECG recordings underwent a streamlined preprocessing pipeline designed for low latency. We deliberately avoid computationally intensive reconstruction techniques (e.g., Independent Component Analysis or channel interpolation) to preserve the original signal manifold and ensure the system's viability for real-time deployment. Crucially, regarding artifact handling, we explicitly chose not to remove ocular activities (e.g., blinks and saccades). In the specific context of fatigue detection, these physiological patterns serve as valuable biomarkers rather than mere noise, as extensive research has linked fatigue onset to distinct ocular signatures such as increased blink duration (Naim et al., 2022; Faust et al., 2018; Zheng and Lu, 2017). By retaining these signals, we enable the classifier to leverage inherent fatigue characteristics as informative features. This strategy not only preserves critical physiological information but also avoids the computational overhead of artifact removal algorithms, aligning with our objective of efficient pre-flight screening.

A 0.5–30 Hz bandpass filter is firstly applied to the raw EEG signals to attenuate low-frequency drifts (EOG < 0.5 Hz) and high-frequency muscular artifacts. Following filtration, the Fast Fourier Transform (FFT) is employed to decompose the signals into the four target frequency bands (δ, θ, α, and β). For subsequent feature extraction, to continuously capture the dynamic physiological changes associated with fatigue and maximize the utility of the dataset, a sliding window technique is employed for signal segmentation. Specifically, the EEG signals are segmented into 2-s epochs with a 50% overlap (step size of 1 s). This overlapping strategy is widely adopted in physiological computing to prevent the loss of transient information at window boundaries and to serve as an effective data augmentation technique for enhancing the robustness of machine learning models (Zhang et al., 2023; Mouazen et al., 2025; Falih et al., 2024).

**Figure 7:**
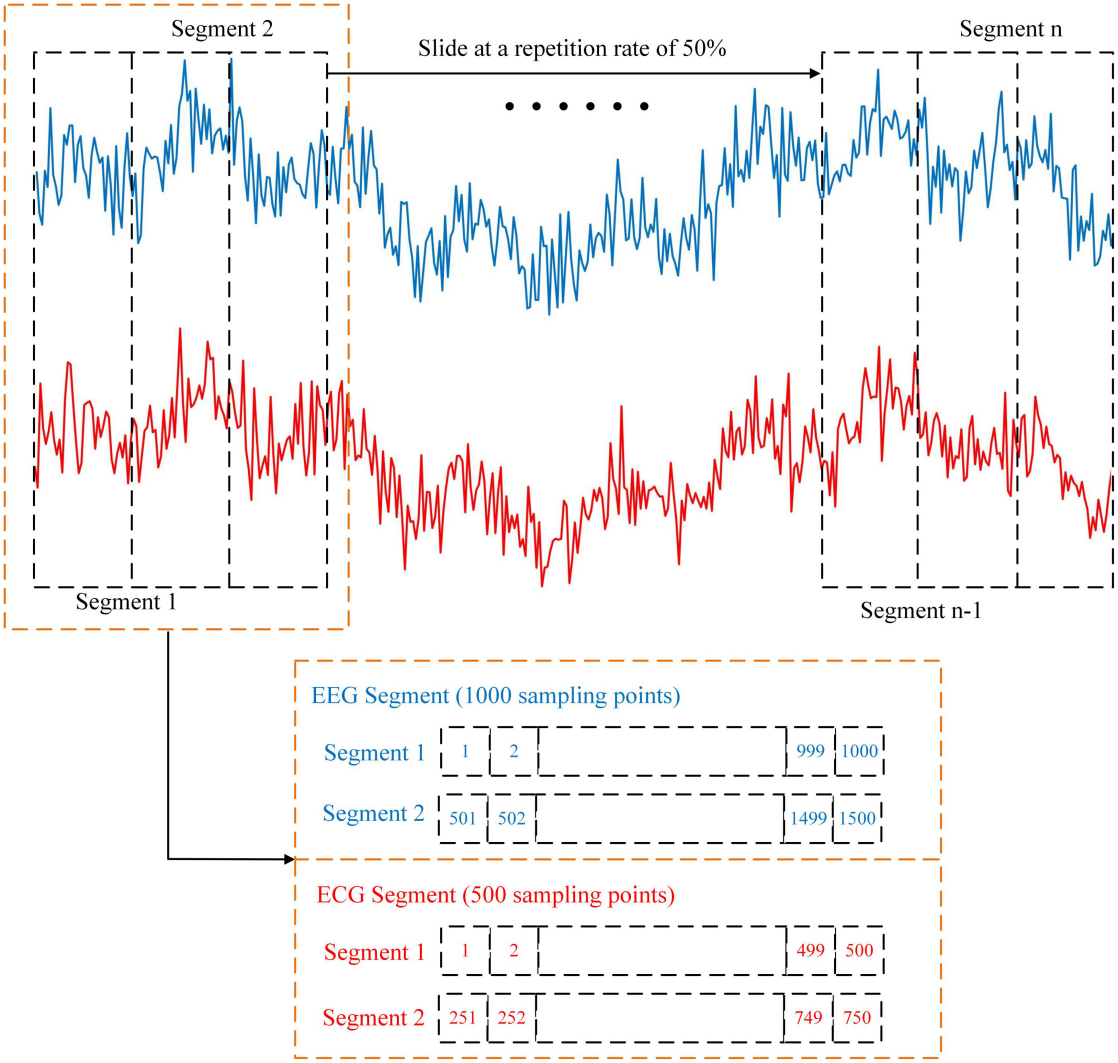
Visualization of the EEG and ECG signal preprocessing and segmentation. Both EEG and ECG signals are filtered (0.5–30 Hz) and segmented into 2-s epochs with a 50% overlap using a sliding window technique. This synchronized segmentation ensures that each data sample contains aligned multimodal information.

The preprocessing of the ECG signals is relatively streamlined. Raw ECG signals are processed with the identical 0.5–30 Hz bandpass filter and then segmented by using the same 2-s windowing approach (with 50% overlap) as the EEG data. This synchronized segmentation is crucial, establishing each 2-s epoch as a single, combined-modality sample for the subsequent fatigue state classification, as illustrated in Figure 7.

To justify the segmentation strategy adopted in this framework, we consider the specific operational requirements of fast pre-flight fatigue detection. Recent studies indicate that EEG signals exhibit quasi-stationarity within short intervals (typically 1–4 s), making short-window analysis a robust approach for fatigue detection (Zhang et al., 2023; Mouazen et al., 2025). Consistent with this finding, our previous work also demonstrated that fatigue detection based on 2-s EEG segments can achieve satisfactory performance (Liu et al., 2025). In contrast, standard ECG analysis, particularly frequency-domain Heart Rate Variability (HRV), typically necessitates longer recordings (e.g., 5 min) to ensure metric stability (Chen et al., 2024; Sun et al., 2024). Therefore, rather than relying on traditional HRV measures which are unstable in short windows, our strategy introduces ECG specifically as an auxiliary modality. We focus on extracting statistical morphological features from these 2-s aligned ECG segments to complement the EEG data. This multimodal fusion strategy aims to leverage the complementary information of ECG to enhance detection accuracy, a hypothesis that is subsequently validated in Section 3.

### 2.4 Physiological feature extraction

Following data preprocessing, a comprehensive set of multidimensional features is extracted from the segmented EEG and ECG epochs to capture the physiological manifestations of fatigue.

For EEG signals, both time-domain and frequency-domain analyses are utilized to generate 8 distinct features for each 2-s EEG frequency band segment. In the time domain, the mean (MEA) $\bar{X}$, energy (ENE) *X*_*e*_, variance (VAR) *X*_*var*_ and root mean square (RMS) *X*_*rms*_ are calculated to quantify signal amplitude and fluctuation intensity. In the frequency domain, based on the Fast Fourier Transform (FFT), the power spectrum density (PSD) *F*_*psd*_, centroid frequency (CF) *F*_*cf*_, frequency variance (FV) *F*_*fv*_ and mean square frequency (MSF) *F*_*msf*_ are extracted to characterize the spectral energy distribution. Previous studies have already demonstrated the effectiveness of these features (Luo et al., 2019; Wang Y. et al., 2024; Zhou et al., 2023; Wang et al., 2021).

For ECG signals, given the limitations of frequency-domain HRV analysis in short segments, seven statistical features are derived from the raw waveform to quantify autonomic instability. These features include the mean μ, standard deviation σ, variance σ^2^, maximum *Max*, minimum *Min*, skewness γ, and kurtosis κ. As detailed in the Introduction, these statistical moments are considered robust proxies for the morphological complexity and non-Gaussian deviations induced by fatigue-related autonomic dysregulation.

Since the mathematical formulations for these standard features are well-documented in prior physiological computing literature, detailed equations are omitted here for brevity. Interested readers are referred to (Guo et al. 2025), (Luo et al. 2019), Wang Y. et al. (2024), (Zhang et al. 2023), (Zhou et al. 2023), (Wang et al. 2021), and (Butkevičiūtė et al. 2022) for the detailed derivations.

Finally, to mitigate the significant inter-subject variability inherent in physiological signals, a subject-wise normalization strategy was implemented. Specifically, all extracted EEG and ECG features underwent Z-score standardization (*z* = (*x*−*u*)/σ) individually for each pilot. This process ensures that the subsequent classification focuses on relative fatigue-induced shifts rather than absolute baseline differences between subjects.

### 2.5 Pivotal feature and EEG channel selection

Extracting multi-dimensional features from EEG and ECG signals enables a model to capture more comprehensive information about underlying neurophysiological states than the raw signals themselves (Cai et al., 2020). This transformation, however, converts the data from a time-series representation to a high-dimensional feature space, which often introduces significant redundancy. This redundancy is especially problematic in EEG, given the high spatial correlation between adjacent channels. To address this, we implemented a combined strategy for both feature selection and EEG channel selection. This dual approach is crucial for mitigating data redundancy, improving computational efficiency, and minimizing the risk of model overfitting.

#### 2.5.1 Feature selection

A one-way analysis of variance (ANOVA) is employed as the feature selection strategy, as it directly tests and quantifies mean differences between groups with high interpretability. Moreover, its efficacy for physiological data analysis has been previously validated (Zhang et al., 2023). Consequently, using ANOVA, we systematically identify features capable of discriminating between the alert and fatigue states at a high level of statistical significance (*p* < 0.01). This selection process is crucial, as irrelevant or redundant features can severely degrade a model's generalization performance (Cai et al., 2020). Notably, to effectively capture the distinct physiological signatures of brain and cardiac activities while avoiding the interference of high-dimensional redundancy, an independent feature selection strategy is implemented for each modality.

For EEG, given its multi-channel nature, the selection is performed in a channel-frequency manner. The ANOVA is applied individually to each feature extracted from the 4 frequency bands across all 16 channels. This process is utilized to pinpoint specific channel-frequency pairs that are statistically sensitive to fatigue. Conversely, for the single-channel ECG, the ANOVA is directly applied to its statistical features. This rigorous independent selection strategy prevents the high-dimensional EEG data from dominating the feature selection process, ensuring that critical ECG indicators are preserved for effective feature fusion.

#### 2.5.2 EEG channel selection

The primary goal of EEG channel selection is to pinpoint the most informative channels for fatigue detection. An effective selection strategy can significantly reduce model complexity and computational load while also simplifying the practical setup for data acquisition. In this study, we have implemented a method to rank each channel's relevance to fatigue. Specifically, a distinct Support Vector Machine (SVM) classifier is trained for each individual EEG channel, using only its extracted features.The resulting Area Under the Receiver Operating Characteristic Curve (AUC) score for each single-channel classifier served as a quantitative measure of that channel's individual contribution to discriminating fatigue. This methodology is well-supported: SVM is a proven classifier for EEG-based fatigue detection (Wang Y. et al., 2024; Fu et al., 2020; Wang et al., 2020; Subasi et al., 2022), and AUC is a robust and standard metric for binary classification (Lin et al., 2014; Tang et al., 2020). The procedure is detailed as:

(1) Training separate SVM model

The channel selection procedure involves training 16 distinct SVM classifiers, one for each channel. Each classifier is tasked with discriminating between the alert and fatigue states, utilizing the complete feature set (from all 4 frequency bands) associated with that single channel. As a supervised learning algorithm, SVM operates by constructing an optimal separating hyperplane. This boundary is determined by maximizing the margin to the nearest data points (the support vectors) and is mathematically defined as:


$$\begin{array}{l} \text{w}\cdot \text{x}+b=0 \end{array}$$ (1)

where **w** is the weight vector normal to the hyperplane, **x** represents the input feature vector, and *b* denotes the bias term.

Since perfect linear separability is not expected in the feature data, slack variables (ξ_*i*_) are introduced to permit some degree of misclassification. This transforms the task into a soft-margin SVM optimization. The optimal parameters (**w** and *b*) are then found by solving the convex quadratic programming problem:


$$\begin{array}{l} \underset{\text{w},b,\xi }{\min}\frac{1}{2}{||\text{w}||}^{2}+C\overset{N}{\underset{i=1}{\sum }}\xi_{i} \end{array}$$ (2)

subject to the constraints:


$$\begin{array}{l} y_{i}\left(\text{w}\cdot {\text{x}}_{i}+b\right)\geq 1-\xi_{i},\xi_{i}\geq 0 \end{array}$$ (3)

where *N* denotes the total number of feature vectors, **x**_*i*_ is the *i*^*th*^ feature vector, and *y*_*i*_∈{−1, 1} represents its corresponding state label. Additionally, the hyperparameter *C* is a regularization term that governs the trade-off between margin maximization and error minimization, while ξ_*i*_ represent the slack variables that allow for a degree of misclassification.

Once trained, the classification is determined by the decision function *f*(*x*) = **w**·**x**+*b*. The SVM classifies a sample **x**_*i*_ based on the sign of *f*(*x*) (e.g., positive for alert, negative for fatigue), while the magnitude of this value indicates the confidence of the classification.

(2) Selecting valuable channels based on AUC

Following the training of each single-channel SVM, its performance is quantified using the AUC value. In this context, the AUC score acts as a direct measure of a channel's discriminative power. A higher AUC signifies that a channel provides more effective information for separating the alert and fatigue states. Ranging from 0 to 1, the AUC score represents the probability that the classifier will rank a randomly selected positive (alert) sample higher than a randomly selected negative (fatigue) sample. The calculation is performed as follows (Zhang et al., 2023):


$$\begin{array}{l} AUC=\frac{\underset{i\in PositiveClass}{\sum }rank_{i}-\frac{T\left(1+F\right)}{2}}{T\times F} \end{array}$$ (4)

where *T* and *F* denote the total counts of positive and negative samples, respectively, and *rank*_*i*_ signifies the ranking number assigned to the *i*^*th*^ positive sample features.

All EEG channels were subsequently ranked based on their respective AUC scores. Channels yielding high AUC values were identified as more discriminative for fatigue and thus selected for the final classification model. Conversely, those with low scores indicating minimal contribution were discarded. This systematic selection process ensures that the subsequent fatigue recognition model is built using only the most informative channels, thereby reducing data redundancy, lowering the computational burden, and enhancing the potential for robust classification performance.

### 2.6 Fatigue detection based on multimodal physiological features

For the final detection of pilot fatigue, we employed XGBoost, an optimized ensemble algorithm implemented under the Gradient Boosting framework (Chen and Guestrin, 2016). XGBoost iteratively constructs a series of decision trees using an additive strategy, merging them into a single, robust predictive model. Its state-of-the-art performance on tabular data makes it exceptionally well-suited for our complex classification task, which involves a fused set of features from multiple physiological signals.

Given a multimodal feature vector *x*_*i*_ (comprising the selected EEG and ECG features), the model's final prediction ŷ_*i*_ is an additive sum of the predictions from all *K* trees:


$$\begin{array}{l} {ŷ}_{i}=\phi \left(x_{i}\right)=\overset{K}{\underset{k=1}{\sum }}f_{k}\left(x_{i}\right),f_{k}\in F \end{array}$$ (5)

where *f*_*k*_ represents the *k*^*th*^ decision tree, which maps an input feature vector *x*_*i*_ to a leaf weight, and F denotes the function space containing possible decision trees.

This set of trees is learned by minimizing a regularized objective function:


$$\begin{array}{l} L\left(\phi \right)=\overset{n}{\underset{i=1}{\sum }}l\left(y_{i},{ŷ}_{i}\right)+\overset{K}{\underset{k=1}{\sum }}\text{Ω}\left(f_{k}\right) \end{array}$$ (6)

This objective combines a loss function, *l*(*y*_*i*_, ŷ_*i*_), which quantifies prediction error, with a regularization term, Ω(*f*_*k*_), which penalizes model complexity to prevent overfitting.

The regularization term is defined as:


$$\begin{array}{l} \text{Ω}\left(f\right)=\gamma T+\frac{1}{2}\text{λ}\overset{T}{\underset{j=1}{\sum }}w_{j}^{2} \end{array}$$ (7)

where *T* denotes the number of leaves in the decision tree, *w*_*j*_ is the weight of the *j*^*th*^ leaf, and the hyperparameters γ and λ control the penalty on the number of leaves and the strength of the L2 regularization on the leaf weights, respectively.

Training is performed additively, where at each iteration *t*, a new tree *f*_*t*_ is added to fit the residuals from the (*t*−1)^*th*^ iteration:


$$\begin{array}{l} {ŷ}_{i}^{\left(t\right)}={ŷ}_{i}^{\left(t-1\right)}+f_{t}\left(x_{i}\right) \end{array}$$ (8)

XGBoost optimizes this objective function efficiently using a second-order Taylor expansion. Leveraging its sophisticated optimization strategy, XGBoost can effectively learn the complex, non-linear relationships within these fused features, thereby achieving more robust and accurate fatigue recognition than would be possible with a single signal source. Our previous research has also shown the XGBoost's superiority in analyzing pilot physiological signals (Liu et al., 2025).

## 3 Results

### 3.1 Identification of statistically significant features

As described in Section 2.5.1, a one-way ANOVA was conducted on the initial set of eight EEG features and seven ECG features (described in Section 2.4) to isolate features significantly relevant to fatigue.

**Figure 8:**
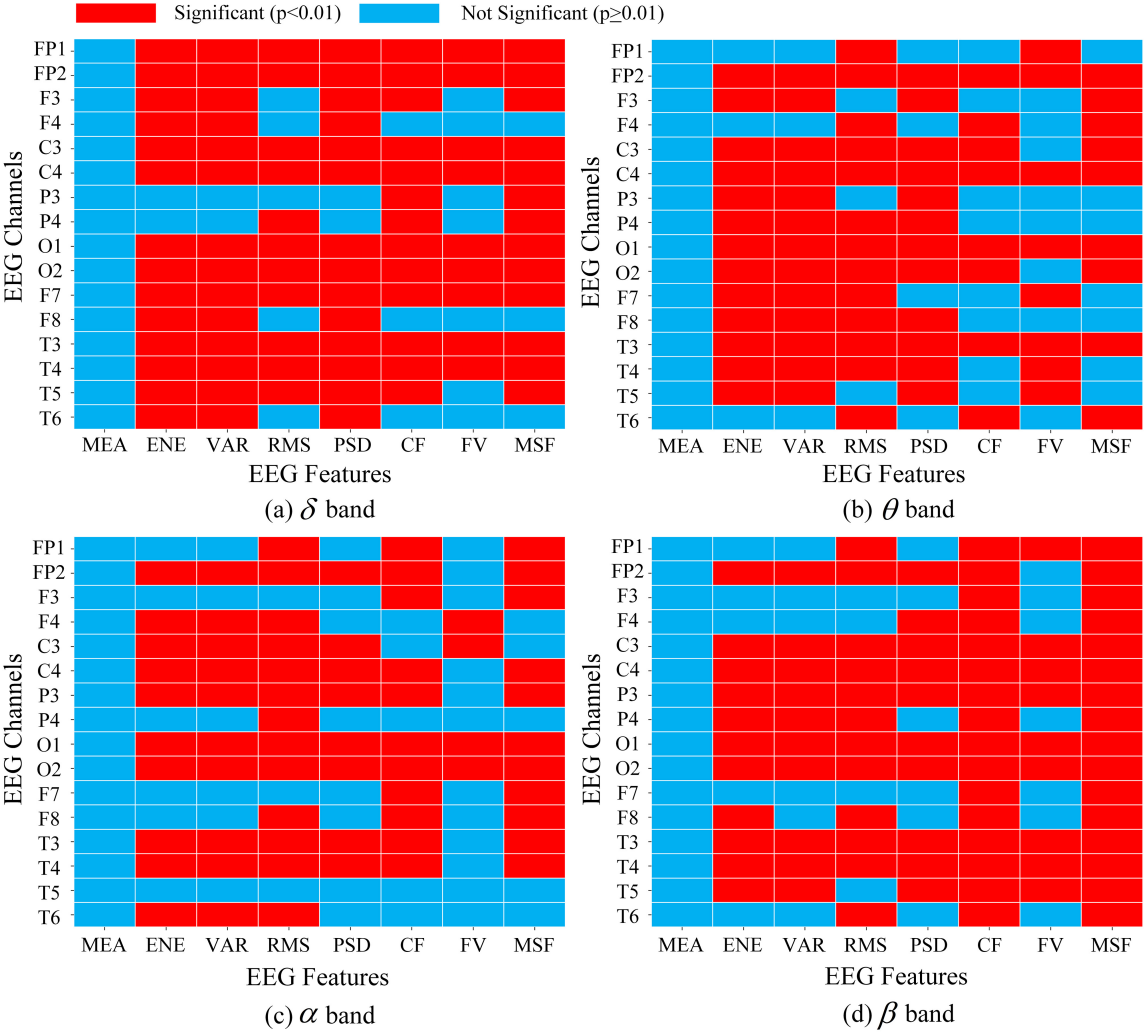
Diagram of ANOVA statistical results of features under alert and fatigue states. The heatmaps display the statistical significance of 8 extracted features across 16 EEG channels for 4 frequency bands: **(a)** δ, **(b)** θ, **(c)** α, **(d)** β. Red blocks indicate significant features (*p* < 0.01) retained for the model, while Blue blocks indicate non-significant features (*p* ≥ 0.01) that are discarded to reduce redundancy.

As shown in Figure 8, the ANOVA results for EEG features revealed varied correlations with fatigue. The majority of frequency-domain metrics (specifically the PSD in θ and β bands) and time-domain features reflecting signal fluctuation intensity (e.g. the ENE, VAR, RMS) exhibited strong statistical significance (*p* < 0.01). These findings are consistent with recent studies, which suggest that these indicators effectively capture the rhythmic neural synchronization associated with fatigue (Zhang et al., 2023; Wang et al., 2025). However, the MEA did not demonstrate statistically significant differences (*p* > 0.05) across most channels. This observation aligns with previous research (Zhang et al., 2023), indicating that the raw mean of EEG signals primarily reflects baseline drifts rather than fatigue-related cortical processing, and thus lacks a robust statistical correlation with fatigue state changes. The FV feature was also deemed unreliable, showing significance across fewer than 10 channels in each frequency band and being removed as well.

For the ECG features, the analysis mainly indicated the superiority of higher-order statistical features over simple central tendency metrics (Table 1). Features characterizing the waveform distribution, such as the σ, σ^2^ and γ showed significant differences (*p* < 0.01). These metrics effectively capture the cardiac instability and autonomic dysregulation associated with mental fatigue. In contrast, the simple μ showed reduced sensitivity (*p* > 0.05), likely due to its susceptibility to individual baseline variations and low-frequency wandering (Guo et al., 2025; Borghini et al., 2014). Overall, only these 4 EEG features were retained: σ, σ^2^, *Max* and γ.

**Table 1:** ANOVA statistical results for ECG features.

**μ**	**σ**	**σ^2^**	***Max***	***Min***	**γ**	**κ**
0.9911	< 0.01(*)	< 0.01(*)	< 0.01(*)	0.5813	< 0.01(*)	0.8743

The *p*-values indicate the significance of the difference between the two states. The symbol (*) denotes statistically significant features with a *p*-value less than 0.01 (99% confidence interval), which represent the most discriminative indicators for detecting pilot fatigue.

These findings demonstrate that not all extracted features provide valid information for fatigue detection. It is necessary to eliminate these non-significant features and retain only the effective physiological indicators.

### 3.2 Evaluation of EEG channel discriminability

As described in Section 2.5.2, AUC was used as the primary metric to quantitatively evaluate each EEG channel's ability to distinguish between alert and fatigue states. The evaluation involved training 16 distinct SVM models, one for each channel, using the 6 salient EEG features identified in Section 3.1. A 10-fold cross-validation strategy was employed to ensure robust performance evaluation.

**Figure 9:**
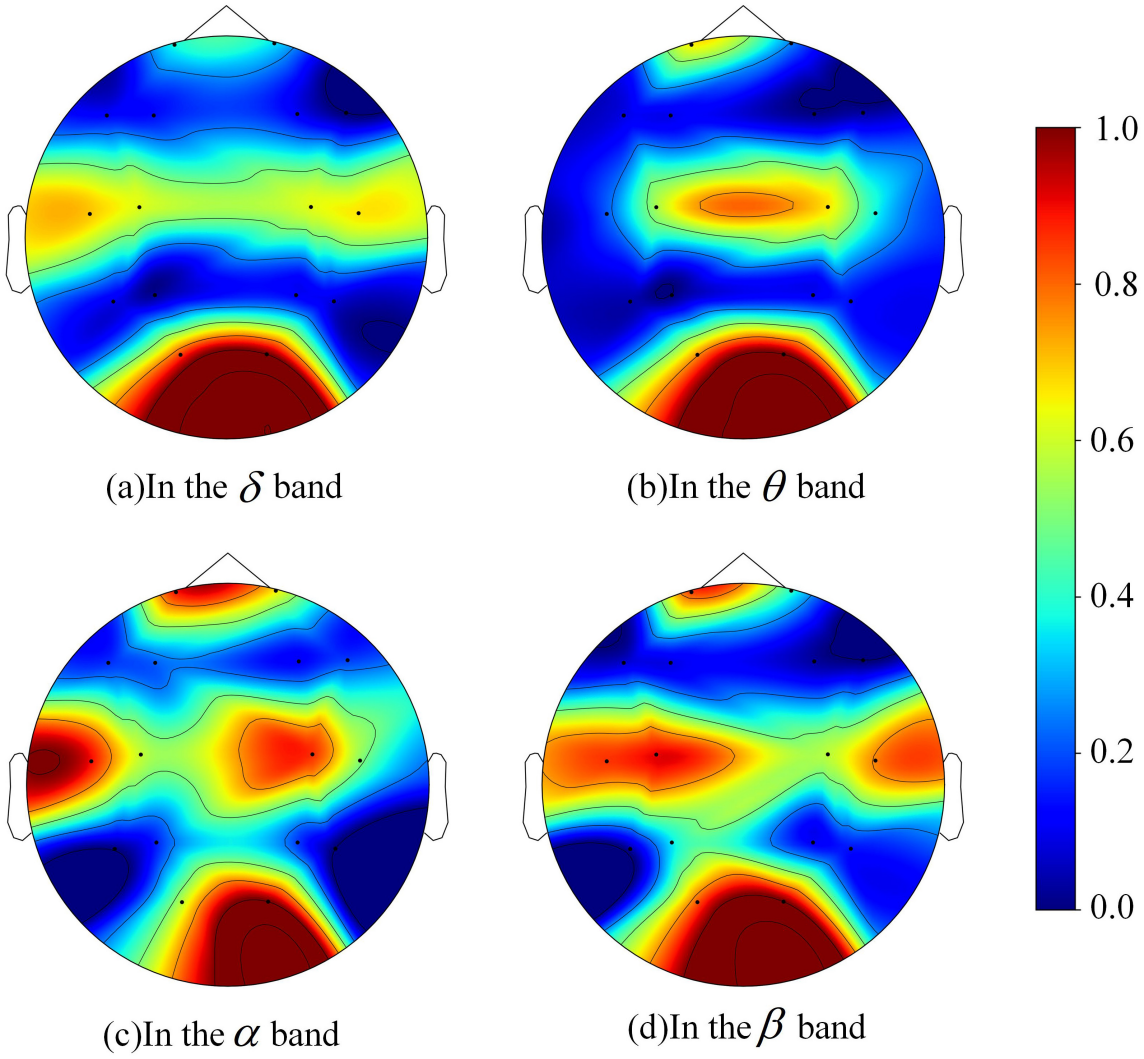
Topographic maps of average AUC values for each EEG channel across 4 frequency bands: **(a)** δ, **(b)** θ, **(c)** α, **(d)** β. The color scale represents the normalized AUC value, ranging from 0 (blue) to 1 (red). Data are obtained based on 10-fold cross-clip cross-validation. With permission from Liu et al. (2025).

The AUC scores were normalized and plotted on brain topographic maps for each frequency band to visualize correlation between each channel and pilot fatigue, as shown in Figure 9. Notably, channels O1 and O2 (occipital region) consistently yielded the highest AUC values across all frequency bands. This finding is physiologically grounded in the nature of pilots, which places heavy demands on the primary visual cortex for instrument scanning and environmental monitoring. Fatigue-induced decline in visual vigilance typically manifests as distinct rhythmic alterations, particularly in alpha activity, within these occipital regions (Jing et al., 2020; Borghini et al., 2014).

Complementing the occipital findings, channels in the frontal (FP1, FP2) and central (C3, C4) regions also demonstrated significant discriminative power. This aligns with the hypothesis that mental fatigue impairs executive functions and attention allocation centered in the prefrontal cortex (Dehais et al., 2019). The significance of the central channels likely reflects the degradation of sensorimotor control required for manipulating the flight stick during prolonged tasks (Jiao et al., 2018). Conversely, channels in the parietal and outer temporal regions consistently demonstrated lower AUC values in this specific experimental setup.

A quantitative summary (Table 2) confirmed these visual findings. A consistent set of eight channels (O1, O2, T3, T4, C3, C4, FP1, and FP2) ranked highest for average AUC, regardless of the frequency band. Since the remaining 8 channels failed to achieve an average AUC above 0.5, this high-performing 8-channel set was selected as the optimal EEG subset for the final model. It is important to note that the maximum mean AUC for a single channel did not exceed 0.65. This apparently low value was an expected consequence of the evaluation method, as each SVM was trained on a minimal feature set (six features from only one frequency band). We hypothesized that performance would improve given more information and this hypothesis was tested by training each channel's SVM model using the EEG features from 4 frequency bands.

**Table 2:** AUC values for single-channel SVM classifiers.

δ **band**		θ **band**		α **band**		β **band**	
**Channels**	**AUC**	**Channels**	**AUC**	**Channels**	**AUC**	**Channels**	**AUC**
**O2**	0.6393	**O2**	0.5945	**O2**	0.5934	**O2**	0.6068
**O1**	0.6172	**O1**	0.5733	**T3**	0.5834	**C3**	0.5989
**T3**	0.5998	**C4**	0.5675	**FP1**	0.5821	**T3**	0.5938
**T4**	0.5956	**FP1**	0.5648	**C4**	0.5815	**FP1**	0.5902
**C4**	0.5904	**C3**	0.5587	**FP2**	0.5625	**O1**	0.5897
**C3**	0.5899	**T4**	0.5445	**C3**	0.5573	**T4**	0.5855
**FP1**	0.5578	**FP2**	0.5328	**O1**	0.5569	**C4**	0.5686
**FP2**	0.5535	**T3**	0.5309	**T4**	0.5527	**FP2**	0.5519
F3	0.5284	T6	0.5244	F3	0.5256	P3	0.5497
F7	0.5276	P4	0.5215	F8	0.5238	T6	0.5302
F4	0.5256	F3	0.5210	P4	0.5234	P4	0.5273
P4	0.5248	F7	0.5208	F7	0.5225	F3	0.5253
T5	0.52262	F8	0.5162	P3	0.5211	F7	0.5247
T6	0.5210	T5	0.5162	F4	0.5178	T5	0.5217
P3	0.5124	F4	0.5143	T5	0.5081	F4	0.5203
F8	0.5101	P3	0.5130	T6	0.5071	F8	0.5172

Each value represents the Area Under the Curve (AUC) obtained by a single-channel SVM classifier using the six key features derived from One-way ANOVA. Higher AUC values indicate a stronger capability of that specific channel to distinguish between alert and fatigue states. All AUC values shown in the table are the averages from 10-fold cross-validation. The bold values indicate the top 8 electrode channels ranked by AUC values for each frequency band. Experimental results demonstrate that these top 8 channels are identical across all frequency bands.

**Figure 10:**
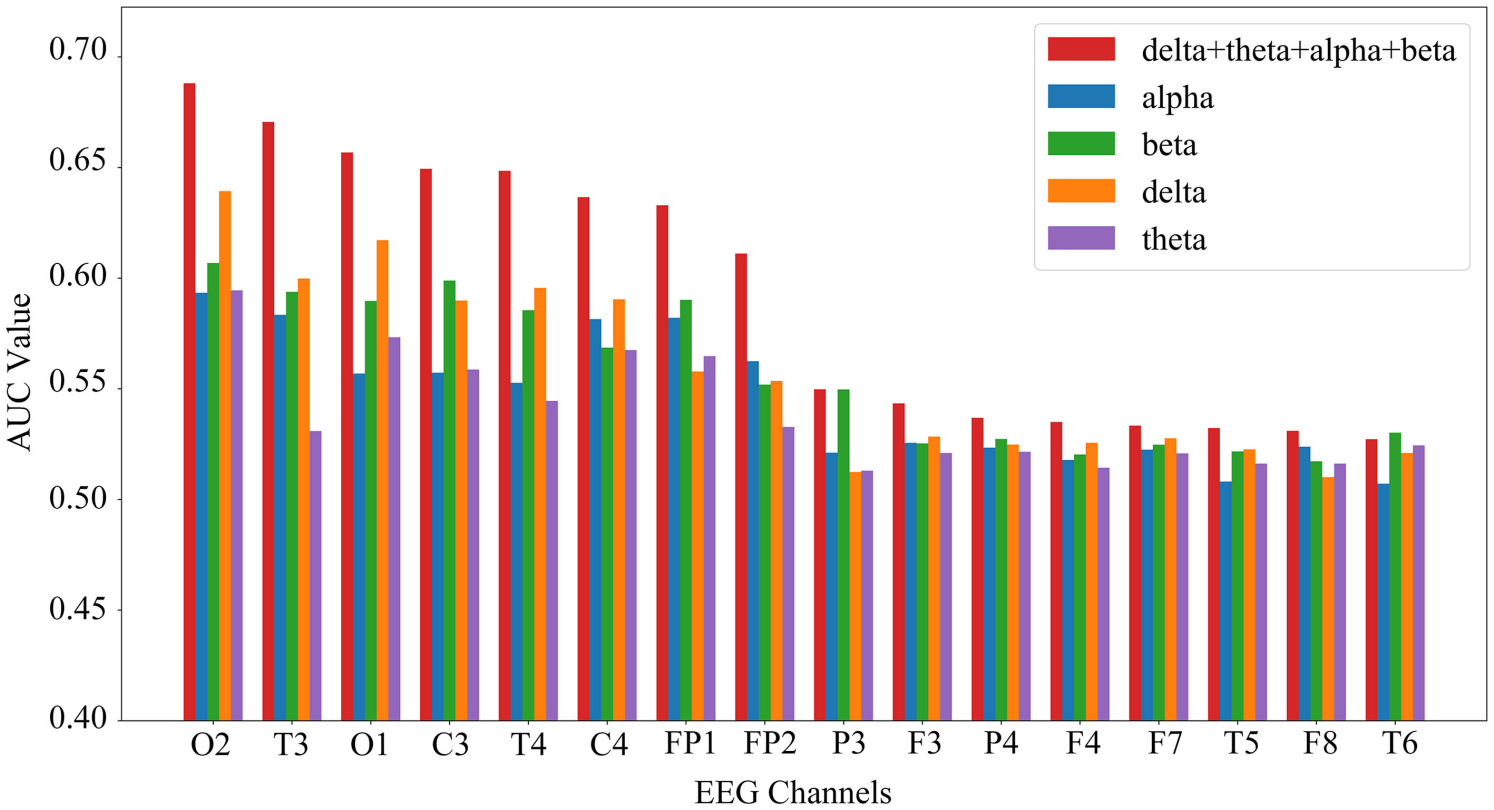
Comparison of single-channel SVM classification performance (AUC) utilizing features from single vs. fused frequency bands. The bar chart compares the AUC of single-channel SVM classifiers using features from individual frequency bands versus fused features from all 4 bands (red bars). Data are obtained based on 10-fold cross-clip cross-validation. Adapted with permission from Liu et al. (2025).

As shown in Figure 10, the results confirmed this hypothesis: for nearly all 16 channels, the AUC achieved using EEG features from 4 frequency bands was significantly higher than that from any single band. The efficacy of this channel selection process is further validated in Section 3.3.3, where the final model's accuracy demonstrates significant improvement.

### 3.3 Performance evaluation

#### 3.3.1 Feature dataset construction

Following the preprocessing (Section 2.3), feature extraction, and channel selection, we constructed the final feature vector for each 2-s epoch. This vector represents a single, independent sample for the classification model. Each vector has a total length of 196 features, composed of the selected EEG and ECG features (4 EEG frequency bands × 6 features × 8 channels + 4 ECG features). This segmentation and feature construction process yielded a final dataset comprising 38,336 samples (32 pilots × 599 epochs × 2 states).

#### 3.3.2 Experimental setup

All experiments were performed in Python 3.10 with PyTorch 2.8.0. The workstation was equipped with an Intel i9-10900X CPU, dual NVIDIA A500 GPUs, and 64 GB of RAM. Hyperparameters for the proposed XGBoost model were set to 100 estimators, a maximum tree depth of 6, and a learning rate of 0.1. For the comparative deep learning models, parameters were set consistent with previous work (Liu et al., 2025): a learning rate of 0.001, a batch size of 64, and 100 training epochs.

All results were evaluated using four standard classification metrics: Accuracy, Precision, Recall, and F1-Score, calculated as follows:


$$\begin{array}{l} Accuracy=\frac{TP+TN}{TP+FP+FN+TN} \end{array}$$ (9)

$$\begin{array}{l} Precision=\frac{TP}{TP+FP} \end{array}$$ (10)

$$\begin{array}{l} Recall=\frac{TP}{TP+FN} \end{array}$$ (11)

$$\begin{array}{l} F1\text{\_}score=\frac{2\times Precision\times Recall}{Precision+Recall} \end{array}$$ (12)

where *TP* (True Positive) is the number of positive samples correctly predicted as positive, and *TN* (True Negative) is the number of negative samples correctly predicted as negative. Conversely, *FP* (False Positive) represents the number of negative samples incorrectly predicted as positive, and *FN* (False Negative) represents the number of positive samples incorrectly predicted as negative.

#### 3.3.3 Detection performances

Model performance was assessed by using two 4-fold cross-validation schemes. The first was a standard cross-clip cross-validation, where all 38,336 samples were shuffled and partitioned into four folds. The second, more stringent scheme was a cross-subject cross-validation, designed to evaluate generalization to unseen individuals, where the samples were divided into four pilot-disjoint folds. To ensure a robust evaluation, this entire cross-subject process was repeated five times with different random grouping.

A systematic comparative experiment was first designed to evaluate the effectiveness of the proposed feature and EEG channel selection method, and verify the complementary role of short-term ECG signals in fatigue detection. Specifically, the XGBoost model's inputs were organized into 6 distinct conditions (Tables 3, 4):

**Table 3:** Ablation study results for XGBoost under different feature input conditions based on 4-fold cross-clip cross-validation.

**Condition**	**Accuracy**	**Precision**	**Recall**	**F1_Score**	**Average training time**
EEG (Baseline)	93.51%	93.47%	93.48%	93.52%	67.6 s
	(±0.32%)	(±0.33%)	(±0.32%)	(±0.32%)	
EEG (w/ FS)	94.08%	94.12%	94.11%	94.07%	62.5 s
	(±0.30%)	(±0.29%)	(±0.30%)	(±0.31%)	
EEG (w/ FS And CS)	96.03%	95.99%	96.01%	96.00%	37.3 s
	(±0.26%)	(±0.27%)	(±0.26%)	(±0.26%)	
ECG (Baseline)	74.88%	74.87%	74.88%	74.89%	5.5 s
	(±0.36%)	(±0.36%)	(±0.37%)	(±0.36%)	
ECG (w/ FS)	77.72%	77.80%	77.72%	77.70%	3.4 s
	(±0.38%)	(±0.38%)	(±0.37%)	(±0.37%)	
**Multimodal fusion**	**98.36%**	**98.37%**	**98.36%**	**98.36%**	39.3 s
	(±0.16%)	(±0.16%)	(±0.16%)	(±0.16%)	

The abbreviations FS and CS denote the Feature Selection and the Channel Selection, respectively. The Baseline conditions utilize the complete feature sets without dimensionality reduction to serve as reference points. The Multimodal fusion condition represents the final proposed framework, integrating the optimized EEG features (subjected to both FS and CS) with the optimized ECG features (subjected to FS). All values are presented as Mean ± Standard deviation. The bold values represent the best performance metrics, achieved across the different experimental conditions, demonstrating the superiority of the proposed multimodal fusion framework.

**Table 4:** Ablation study results for XGBoost under different feature input conditions based on 4-fold cross-subject cross-validation.

**Condition**	**Accuracy**	**Precision**	**Recall**	**F1_Score**	**Average training time**
EEG (Baseline)	80.54%	81.48%	82.49%	81.51%	67.6 s
	(±5.55%)	(±5.61%)	(±6.32%)	(±6.58%)	
EEG (w/ FS)	81.12%	83.21%	80.15%	81.18%	62.5 s
	(±4.31%)	(±6.28%)	(±5.35%)	(±6.32%)	
EEG (w/ FS And CS)	84.02%	84.86%	89.75%	85.98%	37.3 s
	(±3.65%)	(±3.88%)	(±5.89%)	(±5.07%)	
ECG (Baseline)	64.81%	64.75%	66.80%	65.77%	5.5 s
	(±6.85%)	(±6.91%)	(±6.88%)	(±6.89%)	
ECG (w/ FS)	66.76%	66.82%	69.74%	66.78%	3.4 s
	(±6.52%)	(±6.48%)	(±6.55%)	(±6.51%)	
**Multimodal fusion**	**88.42%**	**88.41%**	**88.35%**	**90.18%**	39.3 s
	(±4.53%)	(±4.49%)	(±5.78%)	(±5.51%)	

The abbreviations FS and CS denote the Feature Selection and the Channel Selection, respectively. The Baseline conditions utilize the complete feature sets without dimensionality reduction to serve as reference points. The Multimodal fusion condition represents the final proposed framework, integrating the optimized EEG features (subjected to both FS and CS) with the optimized ECG features (subjected to FS). All values are presented as Mean ± Standard deviation. The bold values represent the best performance metrics, achieved across the different experimental conditions, demonstrating the superiority of the proposed multimodal fusion framework.

(1) EEG Features (no selection): The complete set of EEG features extracted from all channels was utilized as the raw baseline, without the application of any dimensionality reduction techniques.

(2) EEG Features with feature selection only: The proposed feature selection algorithm was applied to the raw EEG dataset. This scenario is established to facilitate a comparison with Condition (1) to evaluate the impact of mitigating feature redundancy.

(3) EEG Features with both feature and channel selection: Both feature selection and the proposed channel selection strategy were implemented. This configuration is intended to isolate the specific contribution of channel optimization by comparing the model performance against Condition (2).

(4) ECG Features (no selection): All extracted ECG features were employed to establish a raw baseline for cardiac physiological activity.

(5) ECG Features with feature selection: The proposed feature selection algorithm was applied to the raw ECG dataset. This scenario is established to facilitate a comparison with Condition (4) to evaluate the effectiveness of the feature selection strategy in mitigating redundancy within the ECG signals.

(6) EEG Features with feature and channel selection, and ECG Features with feature selection: The optimized EEG subset (from Condition 3) was integrated with the optimized ECG subset (from Condition 5). This final scenario is configured to examine the synergistic effects of multimodal fusion and to determine whether the inclusion of ECG signals yields performance improvements relative to the optimal unimodal EEG baseline (Condition 3).

The results clearly demonstrate the efficacy of our proposed optimization methods. For EEG-only data, the fully optimized model (Condition 3) achieved the highest accuracy, while the unoptimized model (Condition 1) performed the worst, confirming our selection process enhances performance. A similar benefit was seen for ECG-only data (Condition 5 vs. 4). Most importantly, the model using the final fused feature set (Condition 6) achieved the highest overall performance, with average accuracies of 98.36% (cross-clip cross-validation) and 88.42% (cross-subject cross-validation). This result validates the effectiveness of our multimodal fusion and the entire proposed framework.

To benchmark this performance, we compared our optimized XGBoost model against several established classifiers for tabular data. This included deep learning models (FT-Transformer, ResNet, MLP), classic machine learning (SVM, LR, KNN), and our previous EEG-only framework [ASFT-Transformer (Liu et al., 2025)]. The FT-Transformer is widely regarded as a state-of-the-art model for tabular data (Gorishniy et al., 2021; Vaswani et al., 2017). Comparative results are shown in Table 5 (cross-clip cross-validation) and Table 6 (cross-subject cross-validation).

**Table 5:** Performance comparison of classification models based on 4-fold cross-clip cross-validation.

**Method**	**Accuracy**	**Precision**	**Recall**	**F1_Score**	**Average training time**
XGBoost	98.36%	98.37%	98.36%	98.36%	39.3 s
	(±0.16%)	(±0.16%)	(±0.16%)	(±0.16%)	
ASFT-Transformer	97.24%	97.25%	97.25%	97.24%	8 min 38.8 s
	(±0.27%)	(±0.27%)	(±0.27%)	(±0.29%)	
FT-Transformer	98.68%	98.68%	98.69%	98.65%	10 min 1.5 s
	(±0.21%)	(±0.21%)	(±0.20%)	(±0.26%)	
ResNet	97.87%	97.88%	97.87%	97.88%	6 min 25.8 s
	(±0.62%)	(±0.61%)	(±0.69%)	(±0.61%)	
MLP	96.06%	96.08%	96.09%	96.03%	3 min 21.7 s
	(±0.29%)	(±0.30%)	(±0.29%)	(±0.29%)	
SVM	85.39%	85.39%	85.34%	85.45%	8 min 7.6 s
	(±0.39%)	(±0.38%)	(±0.34%)	(±0.39%)	
LR	73.49%	73.13%	73.18%	73.30%	15.2 s
	(±0.33%)	(±0.28%)	(±0.27%)	(±0.34%)	
KNN	72.84%	73.66%	72.93%	72.58%	34.7 s
	(±0.51%)	(±0.44%)	(±0.52%)	(±0.55%)	

The performance is evaluated using 4-fold cross-subject cross-validation to assess generalization capability. The comparison metrics include Accuracy, Precision, Recall, and F1-Score, presented as Mean ± Standard deviation.

**Table 6:** Performance comparison of classification models based on 4-fold cross-subject cross-validation.

**Method**	**Accuracy**	**Precision**	**Recall**	**F1_Score**	**Average training time**
XGBoost	88.42%	88.41%	88.35%	90.18%	39.3 s
	(±4.53%)	(±4.49%)	(±5.78%)	(±5.51%)	
ASFT-Transformer	87.72%	87.59%	91.27%	88.94%	8 min 38.8 s
	(±3.76%)	(±4.26%)	(±5.75%)	(±6.60%)	
FT-Transformer	90.58%	90.03%	93.98%	92.15%	10 min 1.5 s
	(±4.01%)	(±4.55%)	(±5.33%)	(±6.99%)	
ResNet	85.77%	85.35%	88.95%	88.41%	6 min 25.8 s
	(±6.32%)	(±5.49%)	(±7.59%)	(±6.11%)	
MLP	82.35%	81.86%	85.83%	83.18%	3 min 21.7 s
	(±3.88%)	(±4.68%)	(±6.57%)	(±6.24%)	
SVM	74.88%	73.08%	78.52%	75.14%	8 min 7.6 s
	(±4.91%)	(±4.33%)	(±7.21%)	(±5.98%)	
LR	67.23%	65.86%	70.18%	67.87%	15.2 s
	(±3.92%)	(±4.59%)	(±5.81%)	(±4.16%)	
KNN	63.89%	63.15%	66.54%	64.91%	34.7 s
	(±4.81%)	(±3.65%)	(±6.74%)	(±4.11%)	

The performance is evaluated using 4-fold cross-subject cross-validation to assess generalization capability. The comparison metrics include Accuracy, Precision, Recall, and F1-Score, presented as Mean ± Standard deviation.

In the cross-clip cross-validation (Table 5), XGBoost model achieved an impressive average accuracy of 98.36%. While this indicates the model's strong capability to fit the data, it is important to note that this metric may be overestimated due to the correlation between overlapping segments from the same subject. Thus, this result should be interpreted as an upper bound of the model's theoretical performance.

Of greater practical significance is the cross-subject cross-validation (Table 6), which evaluates the model's ability to generalize to unseen pilots. In this rigorous testing scenario, the framework maintained a robust average accuracy of 88.42%. This performance is particularly notable as it surpasses our previous EEG-only ASFT-Transformer (87.72%), confirming that the fusion of ECG statistical features effectively compensates for individual variability. Among the classifiers compared, XGBoost demonstrated the best overall balance between accuracy and efficiency. Although the FT-Transformer achieved a marginally higher accuracy in some metrics, XGBoost provided a highly competitive performance with a drastically reduced computational cost (average training time of 39.3 s vs. about 10 min).

**Figure 11:**
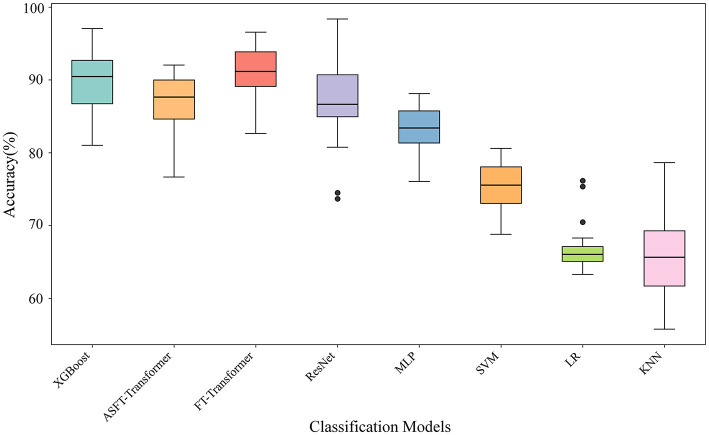
Classification accuracy comparison among different models. The performance is evaluated using 5-time 4-fold cross-subject cross-validation to assess generalization. The central line in each box represents the median accuracy, while the box edges define the interquartile range (IQR). Whiskers extend to the max/min values.

To provide a more detailed analysis of model stability, Figure 11 presents a boxplot comparing the accuracy distributions for each model under the cross-subject cross-validation scheme. This visualization moves beyond the average accuracy reported in Table 6, illustrating the median, interquartile range (IQR), and overall spread of the accuracy scores obtained across the 5 repeated validation runs.

As the boxplot illustrates, our proposed framework based on XGBoost not only achieves a high median accuracy but also exhibits a compact distribution. This indicates highly consistent and robust accuracy when tested on different groups of unseen subjects. While the FT-Transformer reaches a slightly higher median accuracy, our framework's narrow distribution demonstrates comparable stability, reinforcing its reliability. In contrast, several other models show wider distributions, signifying greater variability in their accuracy and less consistent generalization. This visual evidence further substantiates that our framework strikes a balance between achieving high accuracy and maintaining reliable performance.

In summary, while the FT-Transformer achieves the highest absolute accuracy, our proposed framework, by fusing optimized EEG and ECG features, provides an advanced solution. It outperforms our previous EEG-only framework and represents a significant breakthrough in training efficiency, validating it as a solution that strikes an optimal balance between high accuracy, generalization, and computational cost.

## 4 Discussion

This study addresses the need for a pilot fatigue detection framework that balances accuracy with computational efficiency. By transforming physiological signal analysis into a tabular classification task, we successfully integrated optimized EEG and ECG features. The results re-validate the ANOVA-SVM strategy for effective data reduction, which, as shown in Tables 2, 3, lays the foundation for high model performance by eliminating redundant information.

A critical finding is the superiority of multimodal fusion over single-modality approaches. However, regarding validation, a distinction must be made between metrics. While cross-clip cross-validation yielded 98.36% accuracy, this likely overestimates performance due to the correlation of overlapping segments from the same subject. Consequently, we emphasize the cross-subject accuracy of 88.42% as the primary benchmark. This result, derived from unseen pilots, confirms that our framework captures robust, subject-invariant physiological signatures, significantly outperforming the EEG-only ASFT-Transformer (87.72%). Furthermore, the XGBoost classifier reduced average training time to 39.3 s, which is a drastic improvement over the 8–10 min required by deep learning baselines, positioning the framework as a feasible solution for resource-constrained deployment.

## 5 Limitations

To ensure scientific rigor, several key limitations must be acknowledged. First, regarding the labeling strategy, we categorized pre-training and post-training data as alert and fatigue states respectively. This approach was adopted to generate objective labels consistent with literature on prolonged flight tasks and to avoid the variability inherent in subjective self-assessments. However, a limitation remains that without corroborating measures, the physiological state is inferred rather than explicitly validated. Second, the exclusive recruitment of male pilots and the use of a flight simulator limit the ecological validity and generalizability of findings to gender-diverse populations and real-world flight conditions. Third, the use of short-term (10-min) measurements assumes a brief snapshot captures the pilot's state, necessitating further validation in continuous monitoring scenarios. Additionally, regarding statistical analysis, feature selection was performed using independent ANOVAs without correction for multiple comparisons (e.g., Bonferroni or False Discovery Rate). While this heuristic filtering approach theoretically increases the risk of Type I errors, it was chosen to minimize Type II errors (excluding informative features) at the screening stage, with the final feature robustness confirmed by the cross-subject classification performance. In future studies, more rigorous statistical protocols, such as applying correction methods or utilizing multivariate approaches (e.g., MANOVA), are planned to be implemented to further substantiate these physiological findings.

## 6 Conclusion

This paper presents a computationally efficient framework for pilot fatigue detection by fusing multimodal physiological signals. By employing a two-stage optimization process, ANOVA-SVM feature selection followed by XGBoost classification, the study overcomes the computational bottlenecks of deep learning models.

Experimental results indicate the framework's potential for practical application. The system achieved 88.42% accuracy in rigorous cross-subject validation, suggesting that integrating ECG statistical features enhances generalization to new individuals. Coupled with a training time of only 39.3 s, this approach represents an important step toward developing objective fatigue management tools. Future work will focus on addressing the identified limitations by validating this method with diverse populations and subjective fatigue measures in operational environments.

## Data Availability

The raw data supporting the conclusions of this article will be made available by the authors, without undue reservation.
